# Novel migrating mouse neural crest cell assay system utilizing *P0-Cre*/EGFP fluorescent time-lapse imaging

**DOI:** 10.1186/1471-213X-11-68

**Published:** 2011-11-09

**Authors:** Minoru Kawakami, Masafumi Umeda, Naomi Nakagata, Toru Takeo, Ken-ichi Yamamura

**Affiliations:** 1 Division of Developmental Genetics, Institute of Molecular Embryology and Genetics, Kumamoto University, Kumamoto-City, Kumamoto, 860-0811, Japan; 2 Division of Reproductive Engineering, Center for Animal Resources & Development, Kumamoto University, Kumamoto-City, Kumamoto, 860-0811, Japan

## Abstract

**Background:**

Neural crest cells (NCCs) are embryonic, multipotent stem cells. Their long-range and precision-guided migration is one of their most striking characteristics. We previously reported that *P0-Cre/CAG-CAT-lacZ *double-transgenic mice showed significant lacZ expression in tissues derived from NCCs.

**Results:**

In this study, by embedding a *P0-Cre/CAG-CAT-EGFP *embryo at E9.5 in collagen gel inside a culture glass slide, we were able to keep the embryo developing *ex vivo *for more than 24 hours; this development was with enough NCC fluorescent signal intensity to enable single-cell resolution analysis, with the accompanying NCC migration potential intact and with the appropriate NCC response to the extracellular signal maintained. By implantation of beads with absorbed platelet-derived growth factor-AA (PDGF-AA), we demonstrated that PDGF-AA acts as an NCC-attractant in embryos.

We also performed assays with NCCs isolated from *P0-Cre/CAG-CAT-EGFP *embryos on culture plates. The neuromediator 5-hydroxytryptamine (5-HT) has been known to regulate NCC migration. We newly demonstrated that dopamine, in addition to 5-HT, stimulated NCC migration *in vitro*. Two NCC populations, with different axial levels of origins, showed unique distribution patterns regarding migration velocity and different dose-response patterns to both 5-HT and dopamine.

**Conclusions:**

Although avian species predominated over the other species in the NCC study, our novel system should enable us to use mice to assay many different aspects of NCCs in embryos or on culture plates, such as migration, division, differentiation, and apoptosis.

## Background

The neural crest, a pluripotent cell population, produces a variety of cell types, including neurons, glial cells, sympatho-adrenal cells, melanocytes, and mesenchymal cells. Mesenchymal cells in turn form cartilage, bone, and connective tissue. NCCs undergo an epithelial-mesenchymal transition and migrate away from the neural epithelium in streams to different regions of the embryo, where they contribute to the formation of a variety of structures [1]. The processes of NCC induction and migration have been studied extensively [2-4]. Since one of the most striking characteristics of NCCs is the mechanism involving their long-range and precision-guided migration, many studies have focused on this mechanism.

Many molecules have been reported to regulate the migration of NCCs: fibronectin and laminin [5]; collagen [6]; tenascin [7]; chondroitin sulfate proteoglycan (CSPG) [8]; integrin [9,10]; cadherin [11,12]; Eph receptor kinase and their ligands [13]; neuropilin-1 [14-16]; non-canonical Wnt signaling [17]; 5-HT [18]; and PDGF [19-22].

In this study, we focused primarily on cranial neural crest cells (CNCCs), a major component of the vertebrate cranium. Recent experimental observations in mouse, chick, and zebrafish have revived interest in the species-specific aspects of cranial morphogenesis [23-26]. There are still unexplored issues with respect to the molecular mechanisms underlying the patterning and differentiation of NCCs. Each vertebrate species exhibits different patterns of CNCC emigration. For example, in mammals, NCCs begin to emigrate from the tip or 'crest' of the still-open neural folds [27], whereas in birds NCCs arise only after the neural tube closure occurs [28]. Another example of interspecies differences is seen in the pathways of CNCC migration in mammals, which are not nearly as well delineated as they are in birds [29]. On the other hand, fish or frog embryos exhibit markedly different patterns of CNCC emigration from mammals or birds.

Until recently, most studies on CNCCs have been performed on avian embryos because the lineage analysis or direct analysis of NCC differentiation has been hindered in mammals due to a lack of reagents and embryological techniques that allow for the comprehensive characterization of NCCs. Microsurgical manipulation and the *ex-utero *culture of embryos are laborious tasks in most mammals. In addition, a "pan"-NCC cell surface marker, such as the human natural killer-1 (HNK-1) [30], cannot be utilized in mice. Wnt1 is commonly used as an NCC marker in mice [31-33]. However, our purpose is to label NCCs in the mouse head region. Wnt1 does not work for that purpose, because Wnt1 only marks the dorsal neural plate, and labels neuronal cells as well as NCCs, especially in the head region [34]. For all that, in recent years, many NCC studies performed on non-avian model species using new techniques for cell labeling: mouse [35-38]; Xenopus [39-41]; zebrafish [40,42,43]; hagfish [44]; lamprey [45]; and amphioxus [46].

The *P0-Cre *transgenic mouse line is a line that carries a *Cre *gene driven by a *P0 *gene promoter. We previously reported that, by crossing *P0-Cre *mice with *CAG-CAT-lacZ *indicator transgenic mice, expression of lacZ an *E. Coli β*-galactosidase gene) occurs in almost all of the cells and/or tissues that originate with NCCs [47]. In the present study, we used enhanced green fluorescent protein (EGFP) instead of lacZ to observe NCCs in living embryos. By employing a *P0-Cre/CAG-CAT-EGFP *reporter system in fluorescent time-lapse imaging, we demonstrated a novel assay system for mouse NCCs that allows us to observe the behavior of NCCs in real time. This assay system also should facilitate the functional analysis of any factor's effect on NCCs via the implantation of factor-soaked beads. Finally, this assay system should enable assays on mutant mice.

5-HT is a monoamine neuromediator, and it has been shown to control almost every core function of the central nervous system (CNS), such as mood, cognition, sleep, pain, motor function, and/or endocrine secretion [48]. 5-HT is also known as a developmental signal [49]. The agents related to 5-HT (uptake inhibitors, receptor agonists) cause significant craniofacial malformations in cultured mouse embryos. 5-HT was reported to be an important regulator of craniofacial development, and a dose-dependent 5-HT effect on the migration of CNCCs has been demonstrated [18]. However, the molecular mechanisms of this effect have not been characterized very well. Other neuromediators might also affect the migration of NCC. Dopamine is also a monoamine neuromediator and as such is involved in the pathology of movement disorders such as Parkinson's disease or Huntington's disease; it is also involved in psychiatric disorders including schizophrenia [50]. 5-HT and dopamine bind to their specific and respective seven transmembrane receptors, which are coupled with heterotrimeric G protein, and they display many common aspects in their intracellular signaling pathways. 5-HT was reported to reach the mouse embryo at E9 from maternal sources and has been shown to influence development of craniofacial and cardiac mesenchyme [18,51,52]. In the case of dopamine, tyrosine hydroxylase positive cells were reported to be observed in mouse embryos at the medio-basal part of the mesencephalon [53,54] and gut [55] from E10. mRNA of tyrosine hydroxylase gene was observed on E8.5 mouse embryos [56]. These timings of the expression of 5-HT or dopamine overlap with the embryonic stage containing migrating NCCs.

In this study, we isolated GFP-labeled NCC populations from the region rostral or caudal to the midbrain-hindbrain boundary (MHB) of E9.5 *P0-Cre/CAG-CAT-EGFP *embryos, and directly observed single cell migration by utilizing fluorescent time-lapse microscopy. The organizing center, located at the MHB, patterns the midbrain and hindbrain primordia of the neural plate [57] and also affects NCC patterning [58]. We tracked each cell movement in the images, and measured and then summarized the mean migration velocity. We found a difference in the velocity distribution patterns between the two NCC populations. Previous reports demonstrated that 5-HT regulated mouse CNCC migration with modified Boyden chambers [18]. We also assessed the 5-HT and dopamine effects on CNCC migration and found that each agent showed unique dose-dependent and population-dependent patterns of effects on CNCC migration.

## Methods

### Specimens

C57BL/6J mice were purchased from Clea Japan Inc. (Meguro Ward, Tokyo, Japan). Immediately after euthanasia of the pregnant mothers, the embryos were extracted. All animal experiments were carried out with the approval of the Ethics Committee of the Center for Animal Resources and Development, Kumamoto University (D-18-090, A-19-154).

### EGFP Fluorescence Imaging of Embryos

EGFP fluorescence of *P0-Cre/CAG-CAT-EGFP *embryos was detected utilizing a SteREO Lumar V12 fluorescent stereo microscope (Carl Zeiss, Göttingen, Germany).

### Detection of β-Galactosidase (lacZ) Activities

Whole embryos were stained for β-galactosidase activity according to the method of Allen et al. [59]. Samples were stained with X-gal (5-bromo-4-chloro-3-indolyl-*β*-D-galactopyranoside) then fixed in 4% paraformaldehyde/PBS, embedded in paraffin, sectioned to a thickness of 4 μm, and finally stained with hematoxylin and eosin as described by Yamauchi et al. [47].

### Tissue Preparation and Immunohistochemistry

Embryos were fixed in 4% paraformaldehyde/PBS, embedded in paraffin and sectioned to a thickness of 5 μm. Sections were incubated in 3% H_2_O_2 _for 5 minutes, then in blocking solution (10% BSA/PBS) for 20 minutes at room temperature, and then in 1:400 diluted anti-PDGFRα antibody (Santa Cruz Biotechnology, Santa Cruz, CA, USA) overnight at 4°C, followed by a secondary antibody incubation. A Vectastain ABC Kit (Vector Laboratories, Burlingame, CA, USA) was used for the color reaction, and then the embryo sections were counterstained with hematoxylin.

### Mouse Embryo Culture

*P0-Cre/CAG-CAT-EGFP *mouse embryos (E9.0-E9.5) were separated and transferred individually onto a bottom layer of collagen gel (about 2 mm thickness) in two-chamber culture slide dishes (BD Falcon, Franklin Lakes, NJ, USA). The bottom layer was prepared previously from an acid collagen solution (Koken, Toshima Ward, Tokyo, Japan) according to the manufacturer's specified protocol. Embryos were then covered with an approximately 2-mm-thick overlay of the same collagen gel matrix as used in the bottom layer, followed by an overlay of 100% rat serum. These were topped with a mineral oil layer to prevent evaporation. All these structures were incubated at 37°C on a glass heating plate (KM-1; Kitazato Supply Co. Ltd., Fuji, Shizuoka, Japan) under a microscope (SteREO Lumar V12; Carl Zeiss).

### Time-Lapse Imaging of Mouse Embryo Culture

Time-lapse fluorescence images were recorded every 20 minutes for an average of between 24 and 36 hours. Images were digitally collected and analyzed utilizing AxioVision Software and Tracking Module Software (Carl Zeiss).

### Analysis of Cell Migration in Embryos

We chose 20 to 25 cells from the particular NCC population per embryo in the time-lapse images. With the Tracking Module Software, we traced the pathway of migration of each NCC, and analyzed the length of the migration path, elapsed time, and average velocity.

### Bead Implantation Experiment

PDGF-AA (PeproTech, Rocky Hill, NJ, USA) was reconstituted in 10 mM acetic acid to 0.1 mg/ml, and diluted by F-12 medium (GIBCO, Grand Island, NY, USA) to 4 μg/ml. Cibacron Blue 3GA beads (Sigma Chemical, St. Louis, MO, USA) were soaked into PDGF-AA solution for 1 hour on ice. Control beads were soaked in 10 mM sodium acetate diluted by F-12 to the same ratio as the PDGF-AA. These beads were washed once with F-12 with 10% FCS and then were implanted in the embryos.

### NCC Isolation and Culture from *P0-Cre/CAG-CAT-EGFP *Embryos

E9.5 *P0-Cre/CAG-CAT-EGFP *embryos were selected according to their GFP expression. The rostral or caudal part to the MHB of the embryos was excised by fine spring scissors, cut into small pieces, and trypsinized in a DMEM/F-12 medium (GIBCO). Dissociated cells suspended in the medium were filtered to remove debris and seeded on collagen-coated plates. The plates were settled in a standard incubator (5% CO_2_; 37°C) overnight to wait for the cells to attach to the bottom surface.

### Measurement of Migration Velocities of Cultured NCCs

We performed GFP-fluorescent time-lapse microscopy with cultured NCCs utilizing the 'ImageXpress' cell image screening system (Molecular Devices, Sunnyvale, CA, USA), taking an image every 5 minutes. First, we recorded images for 2 hours without factors, then we paused the recording and added a small amount of DMEM/F-12 medium (5 μl) with a factor or with vehicle. We then re-started the recording for 2 more hours. After all the recording was finished, we analyzed the images with MetaXpress software (Molecular Devices) and then compared the migration velocity of the same cell before and after the factor was added. Statistical analyses for Figure Seven, Eight and Nine (Non-repeated Measures ANOVA, Dunnett's test) were performed utilizing an Excel Add-In AOVs0702.xla software http://homepage2.nifty.com/statdograilroad/stat/MyAddIns.html.

## Results

### Observation of NCCs in Living Embryos at Different Stages

**Figure 1:**
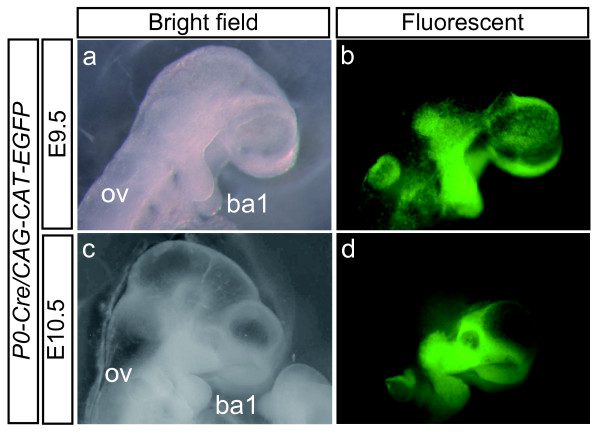
**Detection of Cre Activity in *P0-Cre/CAG-CAT-EGFP *Mouse During Embryonic Development**. E9.5 (a, b) and E10.5 (c, d) embryos. Bright field images (a, c) and corresponding fluorescent images (b, d) (n = 5 each). ba1: branchial arch 1, ov: otic vesicle

Our group reported that by crossing *P0-Cre *mice with *CAG-CAT-lacZ *indicator transgenic mice, lacZ expression occurred in almost all cells of NCC origin in E9.5, E10.5, and E12.5 embryos [47]. In this study, we used a *CAG-CAT-EGFP *strain instead of the *CAG-CAT-lacZ *indicator strain and analyzed fluorescence at E9.5 and E10.5 in living embryos (Figure 1). The distribution of the fluorescence at E9.5 and E10.5 in embryos (Figure 1b, d) was indistinguishable from the lacZ expression pattern of *P0-Cre/CAG-CAT-lacZ *double-transgenic embryos [47]. Next we used the *Rosa26-lacZ *(*R26R*) mouse as an indicator strain. We confirmed that the expression pattern of lacZ is not different from that of *CAG-CAT-lacZ *or that of the EGFP expression pattern of *CAG-CAT-EGFP *(data not shown).

### Time Lapse (Movie)

**Figure 2:**
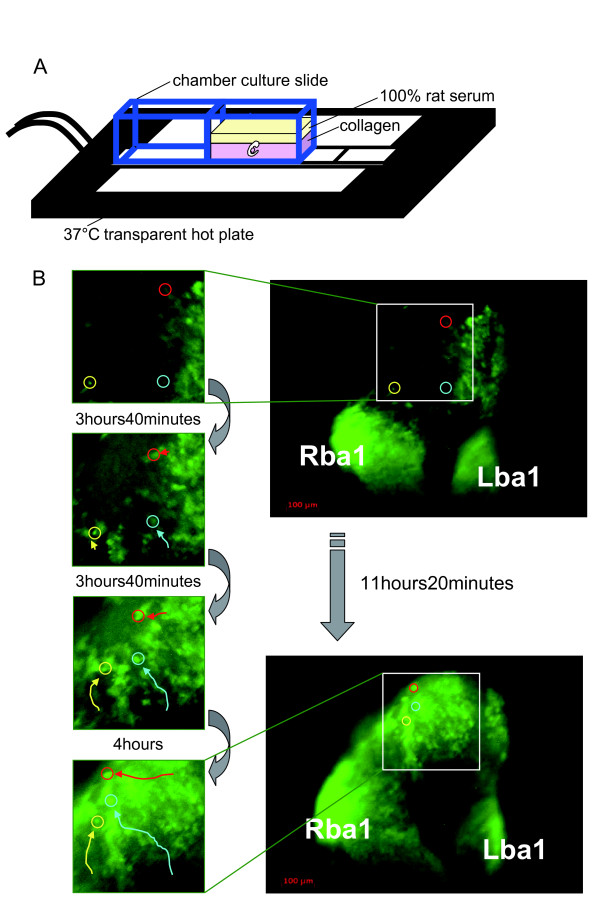
**Migration Patterns of NCCs**. (A) The schematic representation shows the *in vitro *culture system that allowed us to observe. embryos under the microscope. (B) Fluorescent imaging of the cells expressing EGFP. Each figure is an individual frame from a time-lapse movie. The time interval between frames is indicated between the figures. Three differently colored circles (red, yellow, and blue) represent examples of tracing for three distinct migrating NCCs. Rba1: right branchial arch 1, Lba1: left branchial arch 1

To observe the behavior of NCCs in real time under the microscope, we constructed an *in vitro *embryo culture system using chamber culture glass slides (Figure 2A). Similar systems were already reported by other groups [60,61]. This system made it possible to keep embryos developing for more than 24 hours and allowed us to also simultaneously observe the NCC migration patterns. We applied fluorescent time-lapse microscopy to *P0-Cre/CAG-CAT-EGFP *embryos in this system. Our live imaging had high enough resolution to recognize single cells and to thereby be able to trace the movement of each cell. We focused mainly on E9.5 embryos and analyzed CNCC migration using the above-mentioned time-lapse system. Figure 2B shows an example of CNCC migration in an E9.5 embryo.

To analyze cell migration, a vital dye staining method is commonly used, as it is easy and applicable to a variety of cell types. For example, works from Patrick Tam's lab [62,63] and Osumi-Yamashita's lab [64-67] clearly showed that DiI lineage tracing could be used effectively in mouse embryos in combination with whole embryo culture to study mammalian NCC development. Our labeling method utilizes a *Cre-loxP/EGFP *reporter system, in which labeling is both constant and continuous. Patterns of CNCC migration in mice were analyzed in detail with vital dye staining, which Serbedzija et al. pointed out reveals three distinct patterns of CNCC migration [68]: 1) From the level of the forebrain, CNCCs migrated ventrally through the mesenchyme in a sheet, extending from the dorsal part of the neural tube to the level of the optic vesicle, and then they settled in the mesenchyme around the eye. 2) In contrast, CNCCs at the level of the midbrain appeared to migrate ventrolaterally as dispersed cells through the mesenchyme between the lateral surface of the mesencephalon and the ectoderm, toward the general region around the maxillary process or the eye. 3) CNCCs at the level of the hindbrain migrated ventrolaterally, along three segmentally distributed subectodermal streams, from the dorsal portion of the neural tube to the distal portion of the first, second, and third branchial arches. Our data were consistent with the above-mentioned data by Serbedzija et al. [68] for the most part. However, there was an apparent discrepancy concerning the migratory pathway from the forebrain CNCCs. We observed that CNCCs from the level of the forebrain migrated not only through the mesenchyme but also through the ectoderm, and not only in a sheet but also as dispersed cells (Figure 2B, Movie: Additional file 1). They described only one pathway where the CNCCs from the forebrain migrated ventrally through the mesenchyme in a sheet. This discrepancy could be caused by a difference in the method or a difference in the timing of cell labeling. It is also possible that we were able to observe these additional cell populations because our labeling method was continuous and strong.

### Tracking Analysis of NCCs in Embryos

**Figure 3:**
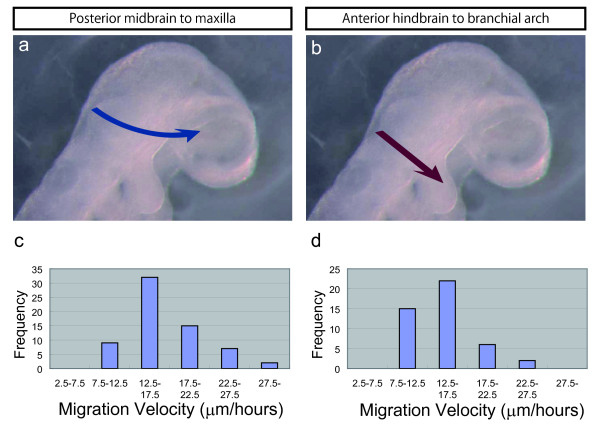
**Tracking Analysis of NCC Migration**. The migration patterns of two closely distributed NCC populations (a, b), and the graphs of the distribution of the migration velocities of NCCs from the two populations (c, d). (a) Population I derived from the most posterior part of the midbrain and migrated. towards the maxillary process and the eye (n = 5). (b) Population II derived from the most anterior part of the hindbrain and went to the first. branchial arch (n = 5). (c) The migration velocity distribution of population I. (d) The migration velocity distribution of population II.

As the manner of CNCC migration differs between the midbrain and hindbrain levels, it is also possible that CNCCs from both levels possess other differences, such as in the mode of "the mean migration velocity" or in "the mean migration velocity" distributions. We compared the migration velocity of these two closely distributed CNCC populations. One population is derived from the most posterior part of the midbrain, and it migrates toward the maxillary process and the eye (Figure 3a). Another population is derived from the most anterior part of the hindbrain and goes to the first branchial arch (Figure 3b). Our time-lapse imagery resolved each CNCC in both population groups. We traced each CNCC movement and then analyzed their mean velocities using Tracking Module software (Carl Zeiss) (Figure 3c, d). We traced an average of 20 cells per embryo from multiple independent embryos. Each of the populations had the mode at the same mean velocity category (12.5 to 17.5 μm/hour) for all of the embryos. On the other hand, there was a difference in "the mean migration velocity" distributions between the two populations. CNCCs from the midbrain level had a broader distribution into the higher velocities compared to those from the hindbrain level (Figure 3c, d). It is likely that this difference in velocity levels reflects the intensity of restraint of each CNCC to the extracellular matrix or adjacent CNCCs in both of the populations, because CNCCs from the midbrain migrate as dispersed cells, as opposed to the CNCCs from the hindbrain, which migrate in streams [68].

### Analysis of Expression Pattern of PDGFRα

**Figure 4:**
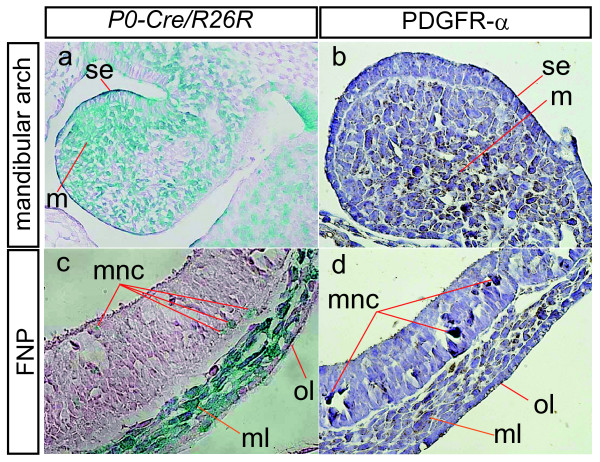
**Comparison of LacZ-Positive Cells in a *P0-Cre/R26R *Embryo and PDGFRα Protein Expression in a Wild-Type Embryo**. E9.5 *P0-Cre/R26R *embryo (a, c) and wild-type embryo (b, d) (n = 5 each). In the first branchial arch, lacZ-positive cells are seen in the surface epithelium and mesenchyme in an E9.5 *P0-Cre/R26R *embryo (a). This distribution shows a strong resemblance to the PDGFRα protein expression pattern seen in an E9.5 wild-type embryo (b). In the frontonasal process in an E9.5 *P0-Cre/R26R *embryo, lacZ-positive cells are seen in the outer layer of the surface ectoderm and mesenchyme tissue layer (c). Separated lacZ-positive cells are scattered in the neuroepithelial wall of the telencephalon. This distribution shows a strong resemblance to the PDGFRα protein expression pattern in an E9.5 wild-type embryo (d). FNP: frontonasal process, se: surface epithelium, m: mesenchyme, mnc: migratory neural crest, ml: mesenchyme layer, ol: outer layer of the surface ectoderm

Several studies have examined the role of the PDGF signaling pathway on CNCCs [19-22,69-71]. *Pdgfra *(platelet-derived growth factor receptor α) mRNA was found to be expressed at high levels in the non-neuronal derivatives of the CNCC, but not in the crest cell neuronal derivatives [20,72]. Because of the lack of an appropriate and adequate NCC marking method, it has been difficult to demonstrate these results unequivocally. In the present study, we compared the expression pattern of PDGFRα analyzed utilizing immunohistochemical staining by a PDGFRα-specific antibody with that of the lacZ expression in *P0-Cre/R26R *double-transgenic embryos at E9.5 (Figure 4a-d). At the mandibular arch or frontonasal process, the PDGFRα expression pattern shows a strong resemblance to the lacZ expression in *P0-Cre/R26R *double-transgenic embryos, although PDGFRα expression was observed not only in NCCs or NCC-derived tissues but also in other types of tissues, such as the paraxial mesoderm and heart (data not shown).

### PDGF-AA Bead Implantation

Mice carrying null mutations in the *Pdgfra *gene have a cleft face phenotype. Although the maxillary process was normal-sized, the frontonasal and mandibular processes were severely reduced in size and unfused at the midline. Most embryos also had a cleft palate and consistently displayed a shortened neck and spina bifida beginning at the cervical level. This phenotype was delineated because of a subset of non-neuronal neural crest cells with high PDGFRα expression that failed to migrate to their proper destinations [20]. PDGFs are known to be involved in chemoattraction, and it is possible that a PDGF-dependent mechanism may play a role in the long-range targeting of CNCC migration [19]. Also, in explant experiments, PDGF-AA enhances NCC motility without affecting the proliferation rate and stimulates cultured NCCs to secrete matrix metalloproteinase 2 (MMP2) and its activator, membrane-type matrix metalloproteinase (MT-MMP) [73]. A few years ago, it was reported that micro-RNA Mirn 140 downregulated the expression of *Pdgfra *in CNCC, and maintained a restricted expression pattern of *Pdgfra*[74,75]. These results also demonstrated the attractive effect of PDGF on NCCs.

**Figure 5:**
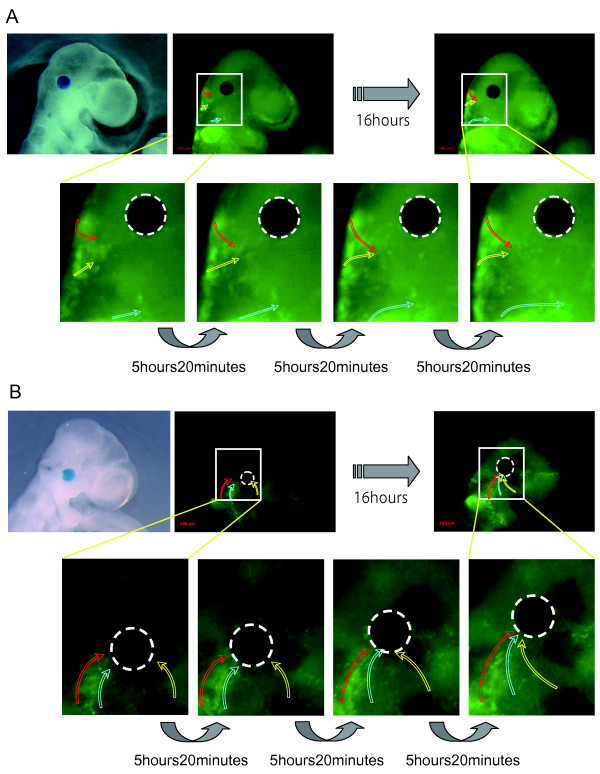
**PDGF-AA Soaked Bead Implantation Affected the NCC Migration**. Fluorescent imaging of the cells expressing EGFP, which shows the migration pattern of NCCs in an E9.5 *P0-Cre/CAG-CAT-EGFP *embryo after the implantation of a control bead (A) or of a PDGF-AA soaked bead (B) in the cranial region. Each figure is an individual frame from a time-lapse movie. The time interval between frames is indicated between the figures. Three differently colored curved arrows (red, yellow, and blue) represent examples of tracing for three distinct migrating NCCs. White circles with dotted lines indicate the position of the implanted bead (n = 5 in each bead implantation).

In the present study, we set out to determine the direct function of PDGF on CNCCs. We implanted PDGF-AA-soaked beads in E9.5 *P0-Cre/CAG-CAT-EGFP *embryos. Fluorescent time-lapse imaging demonstrated that many CNCCs on the surface of the embryos veered off of their original pathway, or even reversed back, and they were obviously attracted to the vicinity of the PDGF-AA beads (Figure 5B, Movie: Additional file 2). This attractant effect was observable within a few hours after the bead implantations, and it was too brief to show any effect on intermediate tissue in order to release any guidance cues for CNCCs. Control beads that were soaked in vehicle did not demonstrate any attractive effects (Figure 5A). This suggested that, at least in our system, PDGF-AA seems to work as a long-range remote attractant to CNCCs in living embryos. However, it is still unclear whether the PDGF signaling in NCCs is necessary for their migration or not, as changes in cell migration were not observed in conditional mutant embryos that lacked PDGFRα in NCCs [21].

### Measurement of Migration Velocities of Cultured NCCs from Different Neural Tube Levels

The MHB region has been known to work as an organizer for anterior neural patterning [57], and it also has been shown to affect NCC patterning [58]. We isolated cells from the region rostral or caudal to the MHB of E9.5 *P0-Cre/CAG-CAT-EGFP *embryos. We named them the forebrain-midbrain NCC (FMB-NCC) for the rostral NCC population and hindbrain NCC (HB-NCC) for the caudal NCC population. We then seeded the isolated cells on collagen-coated plates for 4 hours of time-lapse observation, taking an image every 5 minutes. The *P0-Cre/CAG-CAT-EGFP *system enabled us to distinguish cells with neural crest origin from the other cells by GFP fluorescent microscopy.

**Figure 6:**
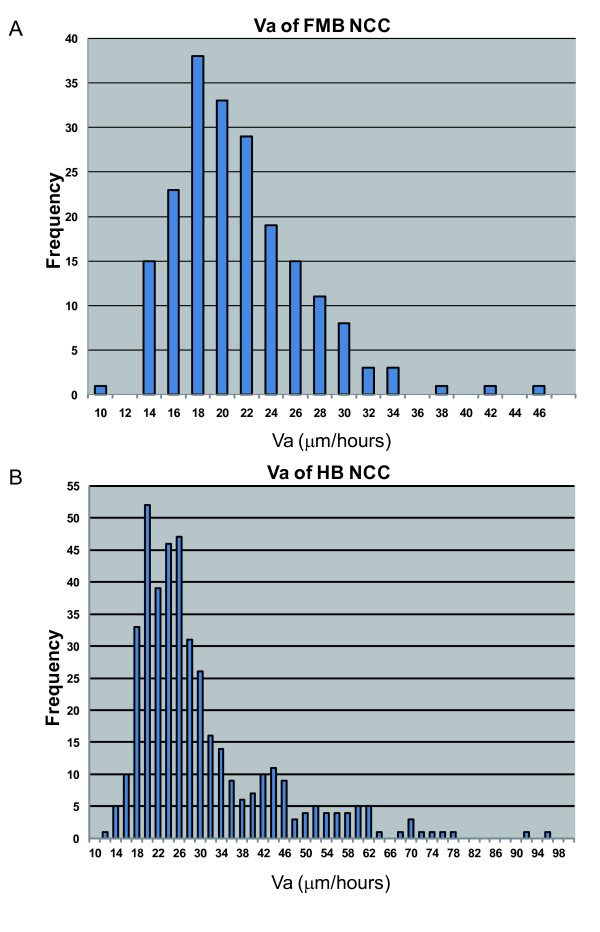
**Comparison of the Distributions of Migration Velocity Between FMB- and HB-NCCs**. E9.5 *P0-Cre/CAG-CAT-EGFP *embryos were used as samples to discriminate NCCs from the other cell types. We isolated cells from the region rostral or caudal to the MHB. We named the rostral NCC population FMB-NCC and the caudal NCC population HB-NCC. Then we seeded the isolated cells to collagen-coated plates for 4 hours of time-lapse observation. We measured the "mean velocity through all the frames (Va)" of both populations (A (n = 201) and B (n = 417)). The two populations have similar Va mode value categories (FMB-NCC: 16-18 μm/hours, HB-NCC: 18-20 μm/hours) and were also similar in minimum Va (FMB-NCC: 9.6 μm/hours, HB-NCC: 11.2 μm/hours). Big differences were observed between the populations in maximum Va (FMB-NCC: 45.5 μm/hours, HB: 94.5 μm/hours), mean Va (FMB-NCC: 20.4 μm/hours, HB-NCC: 29.1 μm/hours), and standard deviation (SD) of Va (FMB-NCC: 5.4 μm/hours, HB-NCC: 13.1 μm/hours).

First, we measured "the mean velocity through all the frames (Va)" of both populations (Figure 6A, B). The two populations have similar Va mode value categories (FMB-NCC: 16-18 μm/hour, HB-NCC: 18-20 μm/hour) as well as similar minimum Va (FMB-NCC: 9.6 μm/hour, HB-NCC: 11.2 μm/hour). Big differences were observed in the maximum Va (FMB-NCC: 45.5 μm/hour, HB-NCC: 94.5 μm/hour), the mean Va (FMB-NCC: 20.4 μm/hour, HB-NCC: 29.1 μm/hour), and the standard deviation (SD) of Va (FMB-NCC: 5.4 μm/hour, HB-NCC: 13.1 μm/hour) between the two populations. Compared to FMB-NCC, HB-NCC had a higher maximum value or SD of Va. The mode categories of Va (FMB-NCC: 16-18 μm/hour, HB-NCC: 18-20 μm/hour) were similar to those of *in vivo *migration (12.5-17.5 μm/hour) (Figure 3c, d).

### *In Vitro *Assay of the Effects of 5-HT and Dopamine on Cultured NCCs

**Figure 7:**
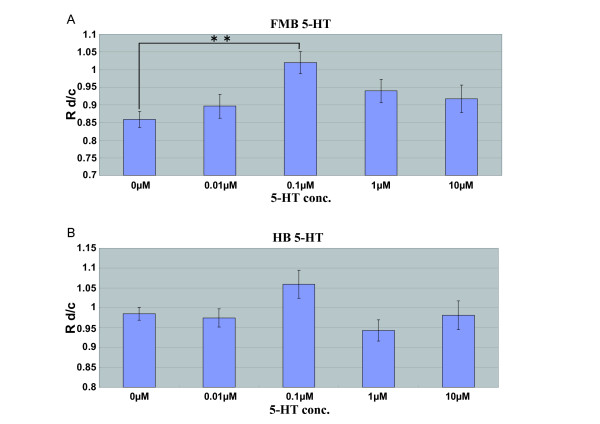
**Dose Dependency of 5-HT's Effect on the Migration Velocity of FMB- or HB-NCCs**. We measured and compared the "mean velocity" of both populations before (Vc) and after (Vd) the addition of 5-HT, and the graph shows the relationship between the Vd/Vc ratio (Rd/c) and each dose of 5-HT (*P < 0.05, **P < 0.01; error bars represent ± s.e.m.). The mean velocity of FMB-NCCs increased after the addition of 5-HT at 0.1 μM (cont: n = 201; 0.01 μM: n = 107, P = 0.798; 0.1 μM: n = 132, P = 0.000101; 1 μM: n = 100, P = 0.176; 10 μM: n = 82, P = 0.521) (A). No increase in the mean velocity of HB-NCCs was observed at any of the concentrations tested after the addition of 5-HT (B) (cont: n = 417; 0.01 μM: n = 214, P = 0.996; 0.1 μM: n = 187, P = 0.126; 1 μM: n = 194, P = 0.617; 10 μM: n = 207, P = 1.00).

We performed *in vitro *fluorescent time-lapse microscopy with or without several different doses of 5-HT or dopamine on cultured NCCs that had been purified from E9.5 *P0-Cre/CAG-CAT-EGFP *embryos. We measured and compared the "mean velocity" of both populations (FMB-NCC and HB-NCC) before (Vc) and after (Vd) upon the addition of either dopamine or 5-HT. Figure 7 shows the relationship between the Vd/Vc ratio (Rd/c) and each dose of 5-HT in the cases of FMB-NCC (A) and HB-NCC (B). The mean velocity of FMB-NCCs was increased after the addition of 5-HT at 0.1 μM (Figure 7A); however, 5-HT did not have any effect on the mean velocity of HB-NCCs at any of the concentrations tested (Figure 7B).

**Figure 8:**
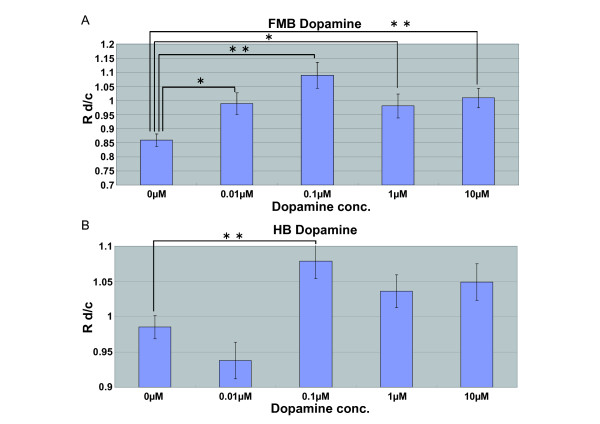
**Dose Dependency of Dopamine's Effect on the Migration Velocity of FMB- or HB-NCCs**. The graph shows the relationship between Rd/c and each dose of dopamine in the case of FMB-NCC (A) and HB-NCC (B) (*P < 0.05, **P < 0.01; error bars represent ± s.e.m.). The "mean velocity" of FMB-NCCs was increased after dopamine addition at all the concentrations tested (cont: n = 201; 0.01 μM: n = 58, P = 0.038; 0.1 μM: n = 78, P = 3.11E-06; 1 μM: n = 70, P = 0.0362; 10 μM: n = 92, P = 0.00169) (A). The mean velocity of HB-NCCs increased after the addition of dopamine at 0.1 μM (cont: n = 417; 0.01 μM: n = 151, P = 0.445; 0.1 μM: n = 236, P = 0.00353; 1 μM: n = 195, P = 0.288; 10 μM: n = 203, P = 0.11) (B).

Figure 8 shows the relationship between Rd/c and each dose of dopamine in the cases of FMB-NCC (A) and HB-NCC (B). Dopamine's effect on the increase in the mean velocity of FMB-NCCs was observed at all the concentrations tested (Figure 8A). As for HB-NCCs, the mean velocity increased after the addition of dopamine at 0.1 μM (Figure 8B).

### *In Vitro *Assay of the Effects of Antagonists on the Stimulatory Effects of 5-HT or Dopamine on Cultured NCCs

**Figure 9:**
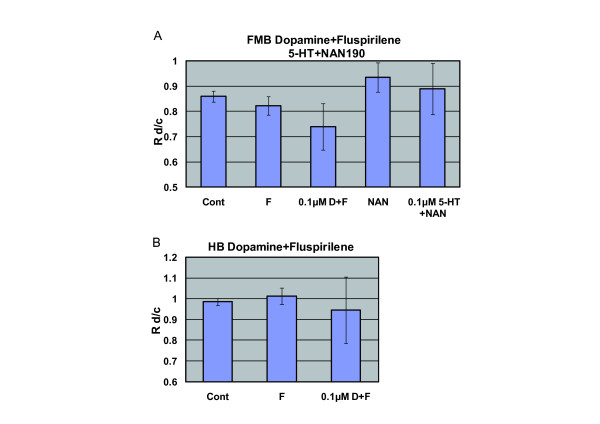
**Effect of 5-HT1A-R Antagonist (NAN-190) or Dopamine D-2R Antagonist (Fluspirilene) with or without the Most Stimulatory Dose of 5-HT or Dopamine on NCC Migration**. The graph shows Rd/c of NCCs treated with antagonists with or without the most stimulatory dose of 5-HT or dopamine. (A) Maximum increase in the mean velocity of FMB-NCCs after the addition of 5-HT was observed at 0.1 μM (Figure 7A). NAN-190 (0.1 μM) markedly decreased the stimulatory effect of 0.1 μM 5-HT, which was reduced to control levels. There were no statistically significant differences between FMB-NCCs after the treatment of antagonist alone (0.1 μM NAN-190) and control in Rd/c (cont: n = 201; 0.1 μM NAN-190: n = 43, P = 0.424; 0.1 μM 5-HT + 0.1 μM NAN-190: n = 39, P = 0.873). In the case of dopamine, the maximum effect was observed at 0.1 μM (Figure 8A). And 30 μM Fluspirilene decreased the stimulatory effect of 0.1 μM dopamine markedly, to control levels. There were no statistically significant differences between FMB-NCCs after the treatment of antagonist alone (30 μM Fluspirilene) and control in Rd/c (cont: n = 201; 30 μM Fluspirilene: n = 43, P = 0.76; 0.1 μM dopamine + 30 μM Fluspirilene: n = 64, P = 0.526). (B) The "mean velocity" of HB-NCCs increased after the addition of dopamine at 0.1 μM (Figure 8B). Fluspirilene (30 μM) decreased the stimulatory effect of 0.1 μM dopamine markedly, to control levels. This amount of Fluspirilene alone caused no significant difference from the control (cont: n = 417; 30 μM Fluspirilene: n = 100, P = 0.757; 0.1 μM dopamine + 30 μM Fluspirilene: n = 17, P = 0.877).

A previous study demonstrated that 5-HTIA receptors are involved in the stimulation of NCC migration. In that report, a 5-HTlA antagonist, NAN-190, inhibited the effect of 5-HT [18]. We therefore tested the effect of NAN-190 on 5-HT. The increase in the mean velocity of FMB-NCCs after the addition of 5-HT was observed at 0.1 μM in the case of FMB-NCCs (Figure 7A). The stimulatory effect of 0.1 μM 5-HT was decreased by 0.1 μM NAN-190 markedly, to control levels (Figure 9A). In the case of dopamine, there are many reports that a dopamine receptor belonging to the D-2 family (D2R) was expressed in the cells derived from NCCs and play roles in regulating the release of catecholamines [76]. D2R belongs to G-protein-coupled receptors and couples to the same type of G protein, Gi, as 5-HT1A [77,78]. It is possible that the stimulative effect of dopamine, as shown in Figure 8A, B, was via D2R. We tested the effect of a D2R-specific antagonist, Fluspirilene, on the effects of dopamine on FMB-NCCs and HB-NCCs. The increase in the mean velocity of FMB-NCCs after the addition of dopamine was highest at 0.1 μM (Figure 8A). In the case of HB-NCCs, the mean velocity increased after the addition of dopamine at 0.1 μM (Figure 8B). All of these stimulatory effects of dopamine were decreased to control levels by 30 μM Fluspirilene (Figure 9A, B).

## Discussion

In this study, we introduced a novel assay system for mouse NCCs employing a *P0-Cre/CAG-CAT-EGFP *reporter system in fluorescent time-lapse imaging. A large number of studies on NCC migration have been performed in the chick, mainly using DiI or electroporation to label the NCCs. The advantage of our genetically engineered reporter system is that, unlike the chick studies, all NCCs are probably labeled. Our technique should help efforts to describe the migration of the minor population of NCCs or to perform long-term observation of NCCs, and should be suitable for use as an assay system. We constructed an *in vitro *culture system for mouse embryos, in which we set the embryos by embedding them into a collagen layer, which made it possible to observe them microscopically by the use of chamber glass slides. This settled-type culture system was necessary for continuous observation via a microscope, though a rotating culture system has often been used prior to this [79]. We used this novel system for 24 to 36 hours of incubation, and we confirmed that most of the embryos kept developing. This system made it possible to examine the localization, migration, and targeting of mouse NCCs with time-lapse images. We measured the migration velocity of mouse NCCs in embryos. We believe that these fundamental data should be very useful for determining the effects of attractive or repulsive factors that affect the long-range targeting of NCCs.

We succeeded in observing the effect of PDGF on the migration of mouse NCCs. This method should be useful for studying other attractive or repulsive factors.

We also measured the migration velocity of isolated mouse NCCs on culture plates. The mode categories of Va of FMB-NCCs and HB-NCCs were 16-18 μm/hour and 18-20 μm/hour, which were similar to those of *in vivo *migration (12.5-17.5 μm/hour). This suggests that the basic characteristics of the migration observed *in vivo *and *in vitro *resemble each other.

On culture plates, we compared "the mean velocity" distribution of FMB-NCC and HB-NCC. The measurement of the mean velocity (Va) of both populations revealed that, compared to FMB-NCC, HB-NCC has a larger maximum value or SD of Va, which means that the migration velocity of HB-NCC had a more prominent positively skewed distribution than that of FMB-NCC. HB-NCC may be made up of more heterogeneous cell populations compared to FMB-NCC.

Many studies have reported that 5-HT regulates craniofacial development [80-86]. In contrast, dopamine is not known to be involved in craniofacial morphogenesis. In this study, we demonstrated for the first time that dopamine has a stimulative effect on the migration of NCCs. Our data do not allow us to reach a conclusion that dopamine plays a role in craniofacial development. Zhou et al. reported the targeted disruption of the mouse tyrosine hydroxylase (TH) gene and presented the phenotypes of embryos carrying homozygous deletion of TH alleles [87]. They showed that inactivation of both TH alleles resulted in mid-gestational lethality. Although they reported that NCCs were among the first TH-positive cells to appear, they did not observe any craniofacial phenotypes in those embryos. About 90% of mutant embryos die between E11.5 and E15.5, apparently of cardiovascular failure [87]. Cardiac NCC, a subpopulation of NCCs, is known to be essential for vertebrate cardiovascular development and *in utero *survival [88-94]. Although it is possible that cardiac NCC was related to the cardiovascular failure of the TH-deficient embryos, we could not find any reports suggesting a relationship between cardiac NCC function and dopamine. Since the downstream signaling pathways of 5-HT1AR and dopamine D2R resemble each other, it is possible that dopamine merely mimics the action of 5-HT. However, dopamine was known to have a function in some tissues originating from NCC [76,95], and our study demonstrated that migrating NCCs responded to dopamine. To prove the role of dopamine in NCC-related morphological events, we are planning to do several experiments with dopamine antagonists or to observe phenotypes of mouse strains that have mutations in genes belonging to dopamine signaling pathways, synthesis pathways, and transporters.

Both FMB-NCCs and HB-NCCs showed responses to dopamine. In contrast, no increase in the mean velocity of HB-NCCs after the addition of 5-HT was observed at any of the concentrations tested, which means that it is highly possible that the stimulative effect of 5-HT reported previously [18] was only for the FMB-NCC; significantly, it might not be stimulative for the HB-NCC. Previous works suggested that 5-HT uptake in the craniofacial region occurred mainly at the epithelia of the developing palate, tongue, nasal septum, and maxillary and mandibular prominences [80,81]. In addition, the selective serotonin reuptake inhibitor (SSRI) Fluoxetine induced abnormality in maxillary, mandibular, and lens vesicles of cultured embryos [83,84]. Although NCCs have some flexibility in their fate even after emergence from the neural tube, in previous reports the mesenchyme around the lens vesicle or inside the maxilla originated from the forebrain and midbrain, and the mesenchyme inside mandible was made from the forebrain, midbrain, and hindbrain [23]. An NCC migration assay with 5-HT by Moiseiwitsch and Lauder [18] was performed using mandibular explants. So our results suggested that the migration of only FMB-NCC but HB-NCC population in the mandible was stimulated by 5-HT.

## Conclusions

All the results of this study demonstrated the usefulness of the *P0-Cre/CAG-CAT-EGFP *reporter system for various NCC analyses. Our *in vitro *embryo culture system is applicable to a variety of embryonic experiments. In an *in vitro *assay under fluorescent microscopy, GFP-labeled NCCs purified from *P0-Cre/CAG-CAT-EGFP *embryos were easily distinguished from cells having other origins; thus, our results were more reliable compared with other methods. Our system enabled us to confirm the effect of 5-HT on FMB-NCC migration, and we newly discovered the effects of dopamine on FMB-NCCs and HB-NCCs. Eventually, this *P0-Cre/CAG-CAT-EGFP *system should become an important tool for various live-cell assays on the nature of NCCs or NCC-derived cells.

## List of abbreviations

CAG: a composite promoter that combines the human cytomegalovirus immediate-early enhancer and a modified chicken beta-actin promoter; CAT: chloramphenicol acetyl transferase; CNCC: cranial neural crest cell; Cre: Cre recombinase from P1 bacteriophage; FMB-NCC: forebrain-midbrain neural crest cell; HB-NCC: hindbrain neural crest cell; HNK-1: human natural killer-1; 5-HT: 5-hydroxytryptamine; *lacZ*: *E. Coli β*-galactosidase gene; loxP: target site for Cre recombinase from P1 bacteriophage; MT-MMP: membrane-type matrix metalloproteinase; NCC: neural crest cell; PDGFR: platelet-derived growth factor receptor; TH: tyrosine hydroxylase; X-gal: 5-bromo-4-chloro-3-indolyl-*β*-D-galactopyranoside

## Authors' contributions

MK designed research. MU performed the embryo culture experiments and histochemistries. TT and NN contributed the maintenance of the mouse colonies. MK performed the assays on culture plates and wrote the paper. KY funded and supervised the project, which was carried out in his laboratory. All authors read and approved the final manuscript.

## Supplementary Material

Additional file 1 **Time-Lapse Movie: An Example of NCC Migration of E9.5 *P0-Cre/CAG-CAT-EGFP *Embryo**. NCCs migrated along the surface of the E9.5 embryo. A *P0-Cre/CAG-CAT-EGFP *embryo over the course of 11 hours and 20 minutes. Their speed was not uniform, and sometimes they were retarded or wandered. Figure 2B shows several frames from a time-lapse movie. Click here for file

Additional file 2 **Time-Lapse Movie: An Example of NCC Migration of E9.5 *P0-Cre/CAG-CAT-EGFP *Embryo with PDGF-AA Bead Implantation**. NCCs migrated along the surface of the E9.5 embryo. A *P0-Cre/CAG-CAT-EGFP *embryo with an implanted PDGF-AA soaked bead (looks black in the movie) over the course of 16 hours. The PDGF bead had a strong attractive effect on the migrating NCCs. Many NCCs ran off the original pathway or even turned back. Figure 5B shows several frames from a time-lapse movie. Click here for file
